# Ten-Year Follow-up of Patients with Epidemic Post Infectious Glomerulonephritis

**DOI:** 10.1371/journal.pone.0125313

**Published:** 2015-05-11

**Authors:** Sergio Wyton L. Pinto, Gianna Mastroianni-Kirsztajn, Ricardo Sesso

**Affiliations:** 1 Division of Nephrology, Hospital Sao Joao de Deus, Divinopolis-MG, Brazil; 2 Division of Nephrology, Federal University of Sao Paulo, Sao Paulo-SP, Brazil; University of São Paulo School of Medicine, BRAZIL

## Abstract

**Background:**

Scarce information on outcomes of epidemic post infectious glomerulonephritis is available. This is a 10-year follow-up of the patients that developed acute glomerulonephritis in an epidemic outbreak caused by group C Streptococcus zooepidemicus in Brazil in 1998, that were also previously evaluated 2 and 5 years after the acute episode.

**Methods:**

In this prospective study 60 cases (out of 134 in 1998) were reevaluated after 10 years, as well as community controls matched by gender and age. They underwent clinical and renal function evaluation, including serum creatinine and cystatin C, estimated glomerular filtration rate (eGFR), albuminuria and hematuria.

**Results:**

Comparisons of clinical and renal function aspects of 60 patients and 48 community controls have not shown significant differences (eGFR <60 ml/min/1.73m^2^ and/or albuminuria >30mg/g creatinine: 13.8% vs. 12.2%, respectively, p = 0.817) except for a higher frequency of hypertension in the cases (45.0% vs. 20.8%, p = 0.009). Comparing the same patients affected in the acute episode, 2, 5 and 10 years later, it was observed an improvement of median eGFR levels at 2 years and a trend toward subsequent stabilization in these levels, associated with decrease in albuminuria and increased hypertension rates in the last survey. At 10 years it was not observed additional reduction of renal function using serum creatinine, eGFR and cystatin C.

**Conclusions:**

During the acute episode of epidemic GN a considerable proportion of patients presented hypertension and reduced renal function; after 2 years and particularly at this 10-year follow-up survey there was no worsening of renal function parameters, except for persistent higher frequency of hypertension. Nevertheless, a longer follow up is necessary to confirm that progressive loss of renal function will not occur.

## Introduction

In Brazil a large outbreak of acute glomerulonephritis (GN) occurred in 1998, associated to unpasteurized milk, when 253 cases of acute GN were reported in the state of Minas Gerais (central Western country region) [1]. Clinical presentation involved fever, headache, and myalgia, followed by cervical adenopathy. After 7 to 10 days, patients developed GN, characterized by oliguria, hematuria, generalized edema, and hypertension, mostly in adults. Nine individuals were initially submitted to renal biopsy that showed diffuse proliferative pattern consistent with post infectious GN. Serologic assays for cytomegalovirus, Epstein-Barr virus, arboviruses (including dengue), coxsackie viruses B1-6, yellow fever, Rocio virus, Andes hantavirus, Puumula virus, and Sin Nombre virus were negative [2]. Throat cultures indicated that GN was associated with Lancefield group C *Streptococcus zooepidemicus*, a cause of bovine mastitis. Epidemiological investigation revealed that patients had consumed a locally produced cheese prepared with unpasteurized milk [3].

Outbreaks of post streptococcal glomerulonephritis (PSGN) have been rare since the 1970s [4] and scarce data on such epidemic glomerulonephritis and especially on their long-term outcomes are available, justifying the present ten-year follow-up report.

## Subjects and Methods

The files of the originally affected patients with their contact addresses and clinical information were kept recorded by the local nephrology team under the supervision of one of the authors (SWP). After 10 years of the outbreak a new clinical survey was conducted and 60 subjects were reevaluated in the present study. In addition, a control group was selected in the community; they were neighbors of the cases and matched to them by age and gender. The controls lived in the region during the outbreak in 1998 and did not have symptoms or signs of the disease at that occasion.

Participants provide their written informed consent to participate in this study, that was approved by the ethics committee of the Universidade Federal de Minas Gerais.

The medical team visited the cases and controls in their homes, collected the clinical information, and obtained early morning blood and urine samples for analyses. During each visit, a recently voided urine sample was collected for dipstick test (Combur 10 Test M; Roche, Mannheim, Germany) and albuminuria assessment; a blood sample was drawn (usually after fasting); an interim history was obtained; and a physical examination was performed. Blood pressure was measured with a mercury sphygmomanometer, while the patient was in the sitting position after 5 minutes of rest. The average of 3 measurements taken with a 1-minute interval was used in the analysis. Hypertension was defined when systolic blood pressure was higher or equal to 140 mmHg or diastolic blood pressure was higher or equal to 90 mmHg, or use of antihypertensive medication. Blood and urine samples were appropriately stored at 4°C and brought to the reference study laboratory. When examined within 1 day, urine samples were kept at 4°C; otherwise, they were frozen at −20°C. Blood samples were examined for serum creatinine (alkaline picrate method) and cystatin C (nephelometry, BN2-Siemens equipment, Siemens Brasil, SP. Serum creatinine was measured in the same laboratory and using the same methodology in all surveys performed since 1998. Albuminuria was assessed by nephelometry with Image-Beckman Coulter (Inc. Diagnostics Division Headquarters, Brea, CA) and values were considered abnormal if greater than 30 mg/g creatinine. GFR was estimated (eGFR) based on serum creatinine (eGFR-creatinine) by the Modification of Diet in Renal Disease (MDRD) study equation, and based on serum cystatin C by the equation eGFR-cys = 76.7 x cystatin C^-1.19^. GFR was considered reduced when lower than 60 mL/min/1.73m^2^.

### Statistical analysis

The chi-square test or Fisher’s exact test were used for the comparison of categorical variables. Student’s t-test or the Mann–Whitney tests (whenever appropriate) were used for the comparison of continuous variables. In the longitudinal analysis that involved eGFR in the years of 1998 (outbreak), 2000 (after 2 years), 2003 (after 5 years), and 2008 (after 10 years) the tests of Friedman and Wilcoxon were applied for numerical variables and the McNemar’s for categorical variables. Analysis of covariance was used to compare mean values of variables adjusted for age and gender. Tests were two-sided and statistical significance was set at p<0.05. The software used was the SPSS v. 15.0.

## Results

Of the 134 confirmed cases of PSGN seen in 1998, 13 died: three in the acute phase of illness (the causes of death were sepsis, cerebrovascular accident and respiratory failure, respectively), six after resolution of the acute illness, with normal renal function (due to congestive heart failure, myocardial infarction, cerebrovascular accident and unknown causes), and four died on chronic dialysis program. Eleven cases required dialysis during the acute phase of illness and 3 of these died within 1 month of disease; 3 recovered renal function and stopped dialysis before 3 months of disease; 5 of them remained on chronic dialysis; of these, four died after more than 2 years on dialysis.

After 10 years of the outbreak, considering the remaining 121 patients some cases could not be located (n = 52) or did not agree to undergo another medical evaluation (n = 9), thus leaving 60 cases for the current study. These cases did not differ at presentation from those not re-examined regarding several characteristics such as: mean age, gender, hospitalization rate, presence of edema, mean systolic and diastolic blood pressure, serum creatinine levels, and need for dialysis. We confirmed that during the follow-up, none of the non-reevaluated cases died or underwent chronic dialysis in the dialysis centers of the state of Minas Gerais.

During the acute phase, renal biopsy was performed in nine patients. All specimens showed “diffuse proliferative glomerulonephritis” as expected in a context of PSGN. No patients received immunosuppressive drug therapy in the acute phase of disease; however, diuretics and anti-hypertensive drugs were frequently used. Immunosuppression including the use of glucocorticosteroids was never prescribed during the entire observation period of 10 years.

Gender and age of patients and controls, as well as blood pressure measurements and laboratory parameters at follow-up are shown in Table 1. Mean systolic blood pressure was significantly higher in cases than in controls. There was no other significant difference between cases and controls in the other variables in this Table.

**Table 1:** Gender, age, blood pressure and renal function parameters by group at 10-year follow-up.

	Cases N = 60	Controls N = 48	P value
Gender			
Males	20 (33.3)	18 (37.5)	0.652
Females	40 (66.7)	30 (62.5)	
Age, years			
	49.0±15.6	43.5±16.5	0.082
Systolic blood pressure, mmHg	130±23	117±16	0.002
Diastolic blood pressure, mmHg	77±19	72±10	0.098
			
Serum creatinine, mg/dL	0.78±0.18	0.81±0.22	0.437
Serum cystatin C, mg/L	0.81±0.26	0.76±0.19	0.283
eGFR-creatinine, mL/min/1.73m^2^	99±27	98±35	0.847
eGFR-cystatin C, mL/min/1.73m^2^	110±34	114±28	0.502
Albuminuria, mg/g creatinine	7.4 (3.7–17.9)	7.0 (4.5–16.1)	0.624

Values are N (%), mean±SD, or median (interquartile range).

After categorization considering the cutoffs that are indicative of altered results, the only variable that was statistically different between the groups was hypertension, whose prevalence was higher among cases than controls (45.0% vs. 20.8%, respectively, p = 0.009) (Table 2). Even after adjusting for age and gender, hypertension remained the only variable that differed between cases and controls, with cases presenting more than twice the prevalence of hypertension than controls.

**Table 2:** Systemic hypertension, and altered renal function tests by group at 10-year follow-up.

	Cases (n = 60)	Controls (n = 48)	P value
Systemic hypertension			
	27 (45.5)	10 (20.8)	0.009
Serum creatinine			
>1.20 mg/dL for females >1.30 mg/dL for males	1 (1.7)	2 (4.3)	0.581
** **Serum cystatin C			
>1.20 mg/L	4 (6.9)	2 (4.4)	0.694
eGFR-creatinine			
<60mL/mim/1.73 m^2^	2 (3.3)	2 (4.4)	0.999
Albuminuria			
>30 mg/g creatinine	7 (12.1)	3 (6.8)	0.508
eGFR-creatinine < 60mL/mim/1.73 m^2^ or albuminuria > 30mg/g			
	8 (13.8)	5 (12.2)	0.817

Systemic hypertension defined as systolic blood pressure ≥140 mmHg or diastolic blood pressure ≥90 mmHg or use of antihypertensive drugs.

Mean±SE levels of systolic blood pressure were more elevated among cases than controls (130±3 mmHg vs. 117±3 mmHg, respectively, p = 0.01), adjusting for age and gender.

In this assessment, the most commonly used antihypertensive medications were angiotensin converting enzyme inhibitors or angiotensin receptor blockers, in 28.3% (n = 17/60) of the cases and 12.5% (n = 6/48) of the controls followed by the diuretic hydroclorothiazide, in 8.3% (n = 5/60) and 8.3% (n = 4/48) of them, respectively. The cases did not report a frequent use of non-steroidal anti-inflammatory drugs or other nephrotoxic drugs. At 5 years of follow-up, angiotensin converting enzyme inhibitors or angiotensin receptor blockers were used by 7 of 56 (12.5%) cases.

Detection of hematuria by dipstick test revealed 11.7% of positivity in the group of patients and 8.4% in the control group (p = 0.812).

The frequency of eGFR-creatinine lower than 60 mL/min/1.73m^2^ did not differ between cases and controls (3.3% and 4.4%, respectively, p = 1.0).

**Fig 1:**
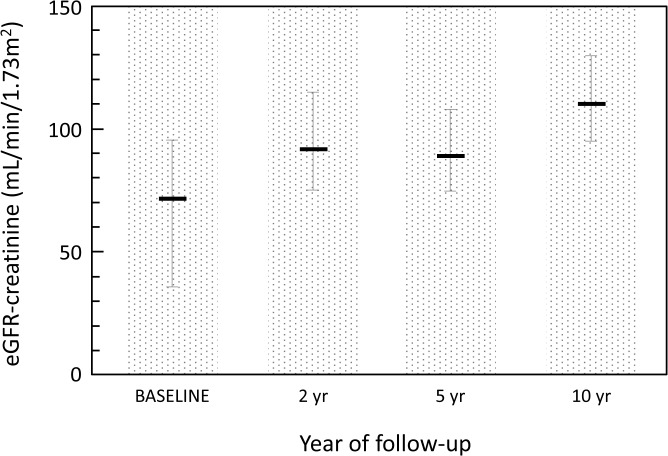
Comparison of the estimated GFR-creatinine values in the cases measured during the follow-up. Values are eGFR-creatinine (mL/min/1.73m^2^) expressed as median (interquartile range (IQR). The overall comparison between groups using the Friedman’s test was significant (p <0.001) and the multiple comparison tests (Wilcoxon’s test with Bonferroni’s correction (p level of significance: <0.020) yielded the following results for the pairwise comparisons: baseline vs. 2 yrs: p<0.001, baseline vs. 5 yrs: p = 0.005, baseline vs. 10 yrs: p<0.001; 2 yrs vs. 5 yrs: p = 0.835, 2 yrs vs. 10 yrs: 0.008; 5 yrs vs. 10 yrs: p<0.001.

In the longitudinal analysis of eGFR-creatinine measurements along the years of follow-up, it was observed a statistically significant difference in the median values, except between the years 2000 and 2003, with an increased median eGFR at the last evaluation compared to previous years (Fig 1).

The analysis using eGFR categorized as ≥ or <60 mL/min/1.73m^2^ showed a higher proportion of patients with lower values in 1998 during the outbreak (42.0%) compared to the three subsequent surveys (p<0.001), but no statistically significant difference was observed between the 2-year, 5-year and 10-year surveys (10.4%, 7.5% and 3.3%, respectively; p = 1.0 for the comparison between 2 yrs vs. 5 yrs, p = 0.063 for the comparison between 2 yrs vs. 10 yrs and p = 0.250 for the comparison between 5 yrs vs. 10 yrs).

None of the patients had nephrotic syndrome. Increased albuminuria (>30 mg/g) was detected in 12% of the cases (n = 6 had values between 30–100 mg/g creatinine and one had 1,633 mg/g creatinine) and 8% of the controls (p = 0.508). Altogether 13.8% of the patients and 12% of the controls (p = 0.817) had increased albuminuria and/or reduced eGFR-creatinine.

## Discussion

*S*. *equi* subspecies *zooepidemicus* is rare in humans but is a well-known cause of disease in cows and horses [5]. In humans, *S*. *zooepidemicus* can cause PSGN [6,7] affecting predominantly adults and other disorders, as sepsis and meningitis [2,8], associated to consumption of unpasteurized milk or milk products [6,7,9] (Table 3).

**Table 3:** Previous reports of outbreaks of acute post streptococcal glomerulonephritis due to *S*. *zooepidemicus*.

Authors	Year of publication	Country	Source of *Streptococcus* *	N
Duca [6]	1969	Romania	Milk	1/3 of GN in 85 cases
Barham, Thorton & Lange [7]	1983	United Kingdom	Milk	3
Francis et al.[9]	1993	Australia	Milk	3
Balter et al.[1] **	2000	Brazil	Homemade cheese	134
Pinto et al.[10] **	*2001*	*Follow-up sample*		*69*
Sesso et al.[3] **	*2005*	*Follow-up sample*		*56*
Present study**	*2014*	*Follow-up sample*		*60*

GN = glomerulonephritis.

*Dairy products have as primary source unpasteurized milk.

** Follow-up approach of the same outbreak (1998).

Re-evaluations of the Brazilian patients of the 1998 outbreak were performed 2 [10] and 5 years later [2,3] and here is presented the 10-year follow-up with the addition of a community control group composed of neighbors of the patients, that lived in the same areas where the outbreak was observed.

It is of note the severe presentation of this outbreak, as well as a high frequency of reduced renal function and/or microalbuminuria, involving almost 50% of the patients 2 years after the acute episode [3,10]. Renal abnormalities persisted in a substantial proportion of patients in the survey 5 years after the onset of the disease (30% had creatinine clearance <60 ml/min/1.73m^2^ or albuminuria >20 μg/min), but after 10 years of follow-up complete recovery occurred in the great majority of cases.

During the follow-up, hypertension and urinary abnormalities were more frequently detected in this series than in other outbreaks of PSGN associated with group A *Streptococcus* [11–14]. In fact the high rates of hypertension along the years of follow-up of our affected population were comparable to those reported previously in epidemic PSGN [14]. In addition, at least two other series of sporadic PSGN in adults have described high rates of hypertension, 29% [15] and 42% [16], respectively, after 2 years of follow-up (Table 4).

**Table 4:** Main findings in the Brazilian outbreak of *S*. *zooepidemicus* leading to PSGN along the years of follow-up.

	N	Hypertension	Reduced GFR*	Microalbuminuria (>20 mg/g creatinine)
**Onset**	134	70%	NA	NA
**2 years**	69 (94% adults)	42%	9%^a^	34%
**5 years**	56 (96% adults)	30%	15%^a^	22%
**10 years**	60	45%	3%^b^	19%^c^

GFR, glomerular filtration rate; NA, not available.

^a^ creatinine clearance 2.

^b^ eGFR (MDRD equation) <60ml/min/1.73m^2^.

^c^12% considering >30 mg/g creatinine.

Additional data on reduced GFR: at onset 62% of the patients had serum creatinine >1.2 mg/dl; 11 patients required dialysis during the acute phase; 5 remained on chronic dialysis.

Few months after the acute episode, it was observed 8% (n = 11/134) of cases with acute renal failure and 3.7% (n = 5/134) with end-stage renal disease, that represent a relatively high rate of loss of renal function in the acute phase of this post infectious GN.

One of the strengths of this study is the use of serum creatinine, eGFR based on serum creatinine and serum cystatin C as additional tools to evaluate more precisely the renal function [17] of this population. Using these markers, considered more sensitive than the absolute levels of serum creatinine to detect reduced GFR, it is possible to assure that a functional recuperation of renal function occurred along the years in the great majority of the patients. High median eGFR rates were observed at the time of the last laboratory assessment. Additional strengths of our report are the prospective study design and the long-term follow-up of this cohort.

As eventual study limitations is necessary to emphasize that the case ascertainment methods used in the earlier phase of such outbreak possibly would not have identified those cases that did not need hospital admission or referral to a nephrologist. Therefore, it is possible that we have studied cases with more severe symptoms and presentation. In addition, despite our efforts, we were not able to reexamine several patients initially affected, however their baseline characteristics were not different from those reassessed and we confirmed that none initiated chronic dialysis in the region during the follow-up. Overall, this sample had a low frequency of risk factors for adverse renal outcomes, as reflected in the very satisfactory renal function results. Also notable is the increasing use of angiotensin converting enzyme inhibitors or angiotensin receptor blockers by the cases (12.5% at 5-yr and 28.3% at 10-yr follow-up), which have contributed to the preservation of kidney function.

Besides the heterogeneity of the studies of PSGN most of them with group A streptococci makes comparison among available reports difficult. Even in group A cases short-term prognosis of GN is undoubtedly good [4], but long-term prognosis is controversial. In general it is considered good in epidemic cases [11,14] and in children [18], and less favorable in medium- to long-term in adults with sporadic GN [12,16,19,20]. Recent studies demonstrate that sporadic PSGN are still a health problem in several regions of the world [21–23]. In some countries as New Zealand there is a significant disease burden of hospitalized acute PSGN with significant short-term complications. On the other hand persistently very low disease rates in European countries, for instance, suggest a preventable disease [23,24].

Especially for its preventable nature, the possible contribution of episodes of acute PSGN to increased risk of chronic kidney disease in later life is a matter of concern. This was investigated in an Aboriginal community in Australia [21]. Of 1519 residents, 200 had had at least one episode of PSGN, usually in childhood. All PSGN episodes were associated with group A streptococcal skin infections, often related to scabies. The authors concluded that PSGN contributes to the very serious burden of chronic kidney disease in that community. They observed, for instance, that PSGN (5 years or more earlier) was significantly associated with higher levels of albuminuria (vs. individuals without previous PSGN) and, in women aged 30–39 years a history of PSGN was associated with a significantly higher frequency of eGFR <60 ml/min.

Post infectious GN including PSGN in their different presentations are current kidney health problems all over the world, as a typical acute PSGN of childhood predominantly in developing countries or involving older adults in the industrialized ones.

Considering our studies and few others it is necessary to be aware to the possibility of *S*. *zooepidemicus* as the etiological agent of acute PSGN, transmitted to humans by unpasteurized dairy products, and affecting mostly adults, as demonstrated in this and other outbreaks.

Long-term reports on epidemic PSGN are scarce, reinforcing the relevance of following during 10 years this population affected by an *S*. *zooepidemicus* outbreak leading to PSGN. In the present study it is noteworthy that after the acute phase, characterized by severe clinical manifestations of this epidemic PSGN (as acute renal failure requiring dialysis, progression to end-stage renal disease, documented crescentic GN), it was observed a trend toward maintenance of renal function instead of progressive loss, especially after 2 years.

Nevertheless, it is still possible that part of these patients have lost a substantial proportion of their nephrons during the acute glomerulonephritis episode and may have reduced nephron mass. The apparent slight improvement in eGFR at 10 years may be partly the consequence of glomerular hyperperfusion/ hyperfiltration injury, particularly considering the increasing percentage of patients with hypertension at this point. Thus, it is still necessary a longer follow-up to dismiss possible deleterious effects of PSGN.
